# Comparative efficacy of two-dimensional mode and color Doppler sonography in predicting gender of the equine fetus

**DOI:** 10.14202/vetworld.2019.325-330

**Published:** 2019-02-23

**Authors:** M. Mebarki, R. Kaidi, A. Azizi, M. Basbaci

**Affiliations:** 1 Higher National Veterinary School, 16270, Street Issad Abbes, Oued Smar, Algiers, Algeria; 2 Veterinary Sciences Institute, University of Blida, BP 270, Algeria; 3 Department of Veterinary Science, Veterinary Sciences and Agricultural Sciences Institute, University of Batna, Algeria

**Keywords:** Doppler mode, fetal sex, mare, two-dimensional mode, ultrasound

## Abstract

**Background and Aim:**

Ultrasonographic fetal sexing is of utmost economic importance for horse breeders. Relatively, a few studies have been conducted to determine fetal sex in mare using transrectal Doppler ultrasound. This study aimed to compare two sexing techniques, two-dimensional (2D) mode and color Doppler ultrasonography.

**Materials and Methods:**

The study was conducted on 39 mares under field conditions. Examinations were performed using the ultrasonic model device (Medison SonoAce Pico, South Korea), equipped with real-time 3-7 MHz convex multifrequency transducer. Fetal sex diagnosis was carried out in two periods of pregnancy, early period (57-80 days of gestation) and late period (80-150 days of gestation).

**Results:**

No difference (p=0.4) was observed between the efficiency of the 2D mode and Doppler ultrasound in sex determination with the respective frequency of 74% and 85%. The best time to sex the early fetus was between 57 and 70 days of gestation and between 90 and 120 days for the older fetus.

**Conclusion:**

The accuracy of sex determination can be increased using Doppler mode compared to 2D mode, especially in the early period between 57 and 70 days, in male more than female sex.

## Introduction

Ultrasound techniques are becoming increasingly important in animal reproduction, by recognizing the disease of the genital tract and monitoring of fetal growth and organ development at various stages of gestation, offering also fetal sex determination technic. Becoming more and more interesting for breeders, fetal gender determination in the mare can provide a useful service. Early sex detection allows implementation of commercial strategies, as the value of the stock at sales time is often determined by the gender of the fetus. It is certain that some horses have a higher proportion of female offspring than males, or vice versa. Moreover, selecting and culling broodmares are easier when the sex of the fetus is known [1]. In contrast, in horses, the choice of desired sex is related to the breed and sportive activity of the animals and often based on the breeder’s individual considerations [2]. In Polo sports, there is a higher demand for the female sex, while in Thoroughbred racing or classical riding competitions the male sex is preferred [3,4].

The first fetal sex determination technic using ultrasound in cow was described in 1986 by Muller and Wittkowski [5]. The fetal gender determination is performed between the 70^th^ and 120^th^ days of gestation, based on visualizing the scrotal swelling in male fetus and mammary glands in female fetus. In 1989, Curran and Ginther [6] had described an early method of fetal sex diagnosis. This technic can be done between the 58^th^ and 70^th^ days of gestation. It is based on the relative position of the genital tubercle, which is initially located on the midline between the hind limbs, making it indistinguishable between males and females fetuses. The genital tubercle starts migration in the 55^th^ day of pregnancy, and it moves a short distance in the caudal direction toward the tail in the female fetus, becoming the clitoris. In the male fetus, the genital tubercle migrates a greater distance in the cranial direction, close to the umbilical cord, becoming the penis. It is easily visualized by ultrasound because it appears in the form of hyperechoic and small bilobulated structure.

However, this technique has some limitations: The examiner has to be experienced to scan the right planes for finding the genital tubercle in both sex and the short period of time it can be performed, which is usually between 59 and 68 days and the evaluation of only one feature of the fetus [7]. Furthermore, in horses, the fetus attached by a long umbilical cord and surrounded by a large amount of allantoic fluid, this allows him to move in the most ventral part of the uterus, making the rectal ultrasound approach extremely difficult and preventing the visualization of its structures [8]. Doppler ultrasound provides a non-invasive and accurate method for the study of blood flow of the female reproductive tract clinically and in research.

There are few studies and data about the use and the efficiency of Doppler ultrasound in gender determination in mare. This study aimed to evaluate the accuracy of equine fetal sex determination using color Doppler ultrasonography in comparison with two-dimensional (2D) mode ultrasound.

## Materials and Methods

### Ethical approval

Ethical approval is not needed to pursue this type of study. However, no mares were harmed during the manipulations. The ultrasonographic examinations were done with the prior consent of the owners.

### Materials

#### Animals

Thirty-nine mares were checked during their gestation, in the period between the 57^th^ and the 150^th^ day. These Arab-barb horses aged from 6 to 15 years belong to private owners. Fetal gender determination was done under field conditions.

#### Ultrasound machine

Examinations were performed using the ultrasonic model device (MedisonSonoAce Pico) with videotape function, equipped with real-time 3-7 MHz convex multifrequency transducer.

### Methods

#### Ultrasonographic evaluation

The mares were restrained in stocks, secured, and handled by horse owners without sedation. First, we had performed rectal emptying from feces without pressing the uterus because any manipulation on this latter can cause a forward displacement of the fetus.

Ultrasound probes are covered with a single-use sheath, after coating the probe with a gel enabling sound transmission, the probe is usually held by thumb, index and middle finger and introduced into the rectum of the mare. A slow scan of the entire uterus was then performed to identify the fetus.

Ultrasound scan was done in two periods of gestation. The early period of sex determination was carried out between the 57^th^ and 80^th^ days of gestation. The area from the umbilical cord to the tail was scrutinized to identify the apparent genital tubercle. If the genital tubercle migrates toward the umbilical cord, it is a male fetus. If it migrates toward the tail, it is a female fetus. Then, the second period or advanced period of gender determination was carried out between the 80^th^ and 150^th^ day of gestation. After locating the fetal head, the probe was gently advanced forward and backward and turned with care to avoid any risk of perforation of the rectum or abortion, until gonad identification. In this period, gender assignment male sex was identified by the presence of the penis and scrotum between fetal hind limbs, while females were identified based on the presence of the vulva and clitoris in the perineal region just under the tail or the mammary glands and their two teats in the ventral abdomen caudal.

First, the examination was performed in 2D mode; then the color Doppler mode was enabled to view and appreciate the detectable blood flow. All ultrasonographic examinations were videotaped to allow later detailed studies and to store images. The time required for diagnosis has been identified. To verify the precision of the determination, data regarding the sex of the foals at birth were collected.

### Statistical analysis

The accuracy of the different ultrasonographic techniques in determining fetal sex at various gestational ages and in the different sexes was compared using the two-sided Fisher’s exact test and odds ratios with their 95% confidence intervals were reported. The level of significance was set to p<0.05.

## Results

**Figure-1:**
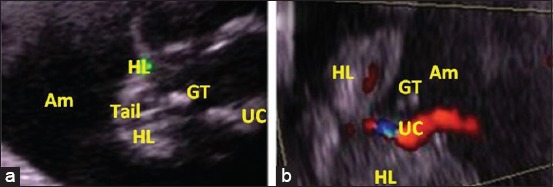
Ultrasound image of a 67-day-old male fetus with two-dimensional mode (a), and color Doppler mode (b). (GT - Genital tubercle, HL - Hind limbs, UC - Umbilical cord, Am - Amnios).

**Figure-2:**
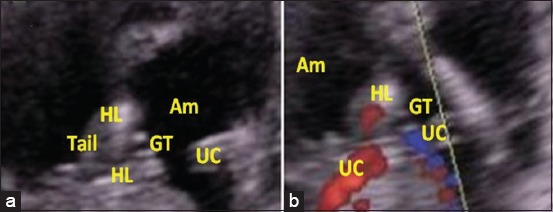
Ultrasound image of a 64-day-old female fetus with two-dimensional mode (a), and color Doppler mode (b). (GT - Genital tubercle, HL - Hind limbs, UC - Umbilical cord, Am: Amnios).

A total of 24 sex determinations were performed between 57 and 70 days of gestation. The identification of the apparent genital tubercle has been scanned by ultrasonographic examination in the area from the umbilical cord to the tail (Figures-1 and 2). In this early period, male fetuses were diagnosed easier due to the larger genital tubercle, with an accuracy of 72.72% against 62.50% in female fetuses (Table-1). Examination of umbilical cord vessels using Doppler ultrasound allows better definition and differentiation from various organs which helps in the examination and easy identification of other organs due to its rich vascularity compared to them (Figures-1 and 2), especially in male 90.90%.

**Table-1:** Fetal sex detection by B mode and color Doppler ultrasonography at various stages of gestation.

Stage of gestation	Male				Female			
	
	57-70 days		90-120 days		57-70 days		90-120 days	
Mode used	B-mode	Doppler	B-mode	Doppler	B-mode	Doppler	B-mode	Doppler
Sex (determined)	8	10	11	12	5	6	5	5
Sex (foaling)	11		14		8		6	
False diagnose	4	1	2	2	3	2	1	1
Accuracy (%)	72.72	90.90	78.57	85.71	62.50	75.00	83.33	83.33

**Figure-3:**
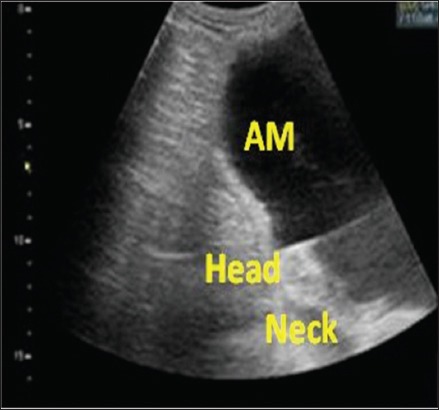
Ultrasonographic image of an 87-day-old undifferentiated fetus. Am - Amnios.

Uterine manipulation from 70 to 90 days of pregnancy during ultrasound examination sinks down the fetus into the abdominal cavity. This makes the fetus less accessible and ultrasound images difficult to interpret because it appears at the bottom of the screen (Figure-3) which prompted us to stop at this period.

**Figure-4:**
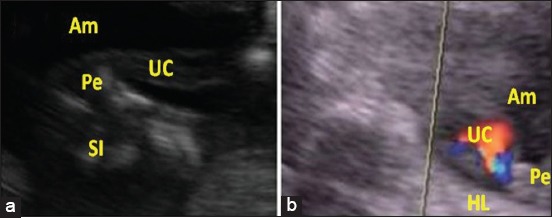
Ultrasound image of a 91-day-old male fetus with two-dimensional mode (a), and color Doppler mode (b). (Pe - Penis, HL - Hind limbs, UC - Umbilical cord, Am - Amnion, SI - Small intestine).

**Figure-5:**
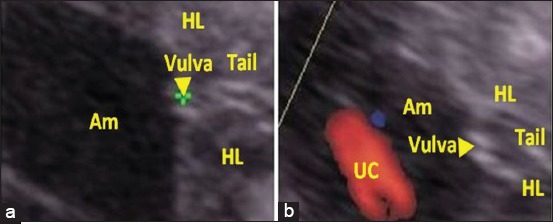
Ultrasound image of a 103-day-old female fetus with two-dimensional mode (a), and color Doppler mode (b). (GT - Genital tubercle, HL - Hind limbs, UC - Umbilical cord, Am - Amnios).

**Figure-6:**
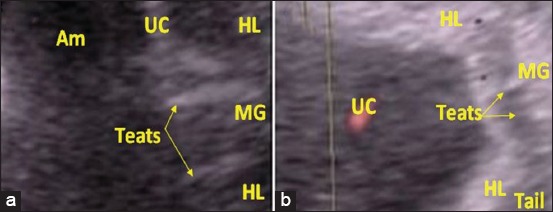
Ultrasound image of a 120-day-old female fetus with two-dimensional mode (a) and color Doppler mode (b). (GT - Genital tubercle, HL - Hind limbs, UC - Umbilical cord, Am - Amnios.

While, between days 90 and 120, only fifteen mares were subjected to sex diagnosis. The area between the two hind limbs was examined for the appearance of the scrotum or the udder. In male fetuses, the genital tubercle will develop into the penis, and its extremity can be visualized using ultrasonography, it appears as a hyperechoic circular bilobulated structure (Figure-4). Moreover, the testes appear located within a scrotum. In the female, the genital tubercle will develop into the clitoris. On ultrasound, it appears as an echogenic and globular mass which can be hard to distinguish from surrounding structures (coccygeal and lumbosacral vertebrae and ischiatic tips of the pelvis) (Figure-5). Moreover, the mammary buds appear using ultrasonography as a highly echogenic bilobed structure (Figure-6).

In this late period, the Doppler mode ultrasonography does not bring more to 2D mode seen the weak vascularization of the examined organs. However, it makes it possible to identify the umbilical cord well and makes it possible to differentiate it from the other organs of the fetus above all the penis which is very close to it in the male (Table-1).

After 120 days, the posterior train of the fetus was positioned beyond the range of the ultrasound probe and becomes quite inaccessible for imaging by transrectal ultrasound.

Mean duration of examination to determine fetal sex between 57-70 days and 100-120 days was about 180 s or less per mare, without counting the rectal emptying time. Time increases to 360 s if diagnosis is made between 90 and 100 days.

The 39 mares gave birth to 25 male foals and 14 female foals. Of the 39 examinations carried out, prenatal diagnosis of the fetal gender was compared with that of newborns: 29 of 39 (74%) using 2D mode and 33 of 39 (85%) using Doppler mode. The frequency of error in sex determination was greater in females than in males.

No difference (p=0.4) is observed between the efficiency of the 2D mode and Doppler in sex determination with the respective frequency 74% (29/39) and 85% (33/39); however, the accuracy of this examination can be increased using Doppler from 74% to 85% (Table-2).

**Table-2:** Accuracy of B mode and color doppler ultrasonography in fetal sex detection.

Mode used	Male		Female		Total accuracy by mode (%)	
		
	B mode	Doppler	B mode	Doppler	B mode	Doppler
Sex (determined)	19	22	10	11	74	85
Sex (foaling)	25		14			
Accuracy (%)	76	88	71	78		
Odds ratio n (95% CI)	0.44 (0.06, 2.39)		0.69 (0.08, 5.25)			
p-value	0.4635		1			

CI=Confidence interval

Overall the period of study, with 2D-mode ultrasonography, the sex was determined in 71% (10/14) of females and 76% (19/25) of males, but this difference was not significant (p=1). With Doppler ultrasound, the sex was determined in 78% (11/14) of females and 88% (22/25) of males. However, the accuracy of this examination can be increased using Doppler in male more than female sex (Table-2).

## Discussion

Transrectal ultrasonography was performed because the determination of pregnancy and fetal sex were easy and fast to obtain using rectal palpation than transabdominal ultrasonography. The period from 57 to 150 days of pregnancy was chosen because there is a consensus that beyond day 150, the fetus becomes inaccessible to transrectal scanning [9]. This wide range interval was chosen to check the efficiency of Doppler in early and advanced pregnancy.

In the present study, mean duration of examination to determine fetal sex was about 180 s or less per mare. According to Livini [10], >85% of all cases were identified within 150 s, and the time to identify fetal sex decreased over the years of study as more experience was gained.

The early period of fetal sexing in the mare is conducted between approximately 57 and 70 days. The technique involves the identification of the genital tubercle, the downward direction of the tubercle is considered a female while the upward direction a male. The migration starts 55 days post-ovulation and can be clearly imaged [10-12]. Literature highlights that the optimal time is between days 59 and 68 [1,6] or 60 and 70 days of gestation [11-14].

After days 70-90, the fetus tends to be placed deeply in the mare’s uterus and becomes quite inaccessible for imaging by transrectal ultrasound and considered by many authors impracticable [1,6,9,11-13], and this supports our findings. A recent study shows that early fetal sexing can be performed between 48 and 75 days of gestation and this extension of the diagnostic window is reliable and profitable in conditions of veterinary practice [15].

The advanced period of fetal gender determination is possible from 90 days to 120 days based on the identification of the prepuce or penis in males or teat, vulva, and clitoris in females. This interval considered as optimum in the late period of fetal sexing [9-12]. After day 120, it was difficult to detect external genital structures of fetus; these results obtained corroborate those of Holder [9], who reported that after 140 days, the fetus is so large and it is difficult to achieve the proper positioning necessary. Renaudin *et al*. [16] observed this problem in advanced pregnancy of >220 days.

Color Doppler ultrasound increases the accuracy of fetal sex diagnosis, but success rates of this technique depend much on skill and expertise of the examiner to rule out potential artifacts and make an accurate diagnosis, consistent with the guidelines of Resende *et al*. [17]. In the early period between 57 and 70 days, our study showed that color Doppler mode has helped us a lot in making the difference between the umbilical cord and the genital tubercle especially in males, which has helped us to perfect the diagnosis. Whereas, in the advanced period of gestation, the Doppler color mode is useless because of the weak vascularization of the genital organs in both: males and females. However, it will be helpful at the stage of 150 to 180 days according to Resend *et al*.[17]. Moreover, Livini [10] found that the best time for sex determination and issue of an official certificate is at 110-130 days of pregnancy when the rate of positive diagnosis is 100%.

Technological advance in the field of large animal research and clinical reproduction allows the emergence of new practical applications of ultrasound in equine fetal sexing. Some studies have shown the use of transrectal Doppler ultrasonography to detect the vascularization of the gonads (pampiniform plexus, testicular vein, and vascular ring between the cortex and the medulla of the ovaries) [17,18]. Others have determined that real-time three-dimensional ultrasonography of equine fetal sex organs also allows obtaining higher quality details of the genital tubercle between days 63 and 76 as well as detailed imaging of the external genitalia structures between days 90 and 150 of pregnancy [19,20].

Furthermore, different techniques of genetic sex identification in embryos before implantation have been developed; these methods are based on cytogenetic analysis and polymerase chain reaction amplification of sex-specific DNA fragments. The major limitation of this method is the fact that it demands well-equipped laboratories and experienced personal [21].

In this study, error in sex determination was more common in females than in males; this result confirms data previously reported by Holder [9]. We have also found that there were no significant differences between the efficiency of the 2D mode and Doppler in sex determination. Our results share a number of similarities with Resende *et al*. [17] findings, that there was no difference between the efficiency of the B mode and Doppler ultrasound techniques for the detection of the male fetuses between 90 and 120 days of gestation and for female fetuses at any of the gestation ages.

## Conclusion

Ultrasonographic fetal sex determination is a rapid, reliable, and feasible technique. The sexing of the fetus is possible during two preferential periods. The early sexing period extends from 57 to 70 day of gestation, with an optimal time between 59 and 68 days. During this period, the diagnosis of sex is based on the position of the genital tubercle in relation to surrounding structures. The late sexing period extends from 90 to 120 days of gestation. During this period, sex diagnosis is based on observation of the external genitalia organs of the fetus. In practice, it is recommended to perform an early sexing of the fetus, but if there is any doubt, a second examination uses to done in the optimum time between 90 and 120 days of gestation.

Fetal sex determination by the transrectal Doppler ultrasonography was an effective technique both in the earlier gestational stage (57-70 days) and the later gestational stage (90-120 days). The time required to determine sex was <180 s, which allows this practice to be employed even at the peak of the breeding season. The accuracy of sex determination can be increased in the early period between 57 and 70 days using Doppler mode compared to 2D mode, especially in male more than female sex. The reliability of the result depends on the conditions of realization (brightness and animal restraint), the setting and quality of the ultrasound system, and the experience of the operator.

## Authors’ Contributions

MM and RK designed the experiment protocol. MM collected the samples and analyzed them. AA was involved in data analysis and scientific reduction. MM, AA, MB, and RK participated in scientific investigations and discussion and drafted the manuscript. All authors have read and approved the final version.

## References

[1] Bucca S (2005). Equine fetal gender determination from mid to advanced-pregnancy by ultrasound. Theriogenology.

[2] Aurich C, Schneider J (2014). Sex determination in horses current status and future perspectives. Anim. Reprod. Sci.

[3] Herrera C, Morikawaa M.I, Belloa M.B, Meyerena M, Eusebio C.J, Dufourqa P, Martinez M.M, Llorentea J (2014). Setting up equine embryo gender determination by preimplantation genetic diagnosis in a commercial embryo transfer program. Theriogenology.

[4] Panarace M, Pellegrini R.O, Basualdo M.O, Belé M, Ursino D.A, Cisterna R, Desimone G, Rodriguez E, Medina M.J (2013). First field results on the use of stallion sex-sorted semen in a large-scale embryo transfer program. Theriogenology.

[5] Muller E, Wittkowski G (1986). Visualization of the male and female characteristics of bovine fetuses by real-time ultrasonics. Theriogenology.

[6] Curran S.S, Ginther O.J (1989). Ultrasound diagnosis of equine fetal sex by location of the genital tubercle. J. Equine Vet. Sci.

[7] Tönissen A, Martinsson G, Otzen H, Schürmann K, Schütze S, Ertmer F, Kassens A, Sielhorst J, Brehm R, Sieme H (2015). To perform fetal gender determination in the mare by ultrasound during early and advanced gestation. Pferdeheilkunde.

[8] Carmo M.T, Oliveira J.V, Almeida M.T, Alvarenga M.A (2008). Avaliaçãoultra-sonográfica da gônada fetal emequinos:Uma nova alternativa para sexagem. In:IX Conferência Anual da ABRAVEQ, 2008. IX Conferência Anual da ABRAVEQ e IV Congress Internacional de Medicina Veterinária, São Paulo.

[9] Holder R.D (2000). Fetal sex determination in the mare between 55 and 150 days gestation. AAEP Proc.

[10] Livini M (2010). Determination of fetal gender by transrectal ultrasound examination:Field's experience. Proc. Am. Equine Pract.

[11] McGladdery A (2011). Equine Fetal Sex Determination. In:Proceedings of the Annual Meeting of the Italian Association of Equine Veterinarians, Montesilvano, Italy.

[12] Turner R.M (2013). Fetal Sexing for the Practitioner. Proceedings of the Annual Resort Symposium of the American Association of Equine Practitioners.

[13] Brinsko S.P, Blanchard T.L, Varner D.D, Schumacher J, Love C.C, Hinrichs K, Hartman D.L, Brinsko S.P, Blanchard T.L, Varner D.D, Schumacher J, Love C.C (2011). Transrectal ultrasonography in broodmare practice. Manual of Equine Reproduction.

[14] Cuervo-Arango J (2015). Choosing the sex of the offspring in a commercial equine embryo transfer center. Med. Weter.

[15] Zuviria S.M, Zagrajczuk A (2016). Application of ultrasound technique in early fetal gender determination - the field study. J. Equine Vet. Sci.

[16] Renaudin C.D, Gillis C.L, Tarantal A.F (1997). Transabdominal combined with transrectal ultrasonographic determination of equine fetal gender during midgestation. Proc. Am. Equine Pract.

[17] Resende H.L, Carmo M.T, Ramires N.C, Alvarenga M.A (2014). Determination of equine fetal sex by Doppler ultrasonography of the gonads. Equine Vet. J.

[18] Resende H.L, Carmo M.T, Alvarenga M.A (2012). Use of doppler ultrasound for equine fetal sex determination. Reprod. Fertil. Dev.

[19] Kotoyori Y, Yoko N, Ito K, Kimura Y, Korosue K, Ashihara N, Nambo Y (2010). Transrectal 3-dimensional ultrasound examination of the equine fetus during the first half of gestation. Anim. Reprod. Sci.

[20] Kotoyori Y, Yokoob N, Itoc K, Murased H, Satod F, Korosued K, Nambod Y (2012). Technical note three-dimensional ultrasound imaging of the equine fetus. Theriogenology.

[21] Crişan M.I, Damian A, Morar I, Páll E, Peştean C, Groza I.Ş (2016). Equine embryo sexing and ultrasonographic fetal sexing interests and applicability. Anat. Histol. Embryol.

